# Supplementary material for: Parental vaccination to reduce measles immunity gaps in Italy
Source: eLife. 2019 Sep 3;8:e44942. doi: 10.7554/eLife.44942 (PMC6721460; doi:10.7554/eLife.44942)
Supplement: Supplementary file 1. [file elife-44942-supp1.zip › elife-44942-supp1-v1/methods_demographic_projections_ITALIAN.pdf]

Nota metodologica

Le previsioni demografiche regionali dell'Istat sono realizzate con l'obiettivo di rappresentare il possibile andamento futuro della popolazione, sia in termini di numerosità totale sia in termini di struttura per età e sesso. Le informazioni prodotte rappresentano uno strumento importante a supporto delle decisioni nelle politiche di natura economica e sociale, come quelle relative ai sistemi pensionistici, sanitari, scolastici e abitativi. Queste vengono aggiornate periodicamente rivedendo e/o riformulando le ipotesi evolutive sottostanti la fecondità, la sopravvivenza, i movimenti migratori internazionali e quelli interni.

Le previsioni in base 2017 sostituiscono quelle in base 2016 pubblicate dall'Istat il 26 aprile 2017. Con ciò l'Istat porta sistematicamente a regime l'aggiornamento annuale delle previsioni demografiche, dopo che per anni la diffusione delle stesse era avvenuta con cadenza perlomeno quinquennale. Rispetto al precedente esercizio previsivo sono stati apportati due cambiamenti: 1) l'aggiornamento della base di popolazione per sesso, età e regione al 1° gennaio 2017; 2) la parziale rivisitazione nel breve termine delle ipotesi demografiche, ricorrendo a strumenti di *nowcasting*, che hanno l'obiettivo di tarare le previsioni demografiche nei primi anni di proiezione sui livelli degli indicatori demografici rilevati nell'ultimo periodo storico disponibile. A parte tali parziali cambiamenti, il resto dell'impianto metodologico è identico a quello utilizzato in occasione del precedente round previsivo, impianto di cui si dà conto sinteticamente in questa nota.

La metodologia alla base delle previsioni è stata definita da un gruppo di lavoro che ha visto la collaborazione di ricercatori dell'Istat, dell'Università Luigi Bocconi di Milano e dell'Università di Oxford. Titolare e responsabile della produzione e della diffusione delle previsioni è l'Istat così come documentato nel Programma statistico nazionale.

Sulla base del contributo emerso dalla collaborazione del gruppo di ricercatori, si è deciso di adottare un approccio di tipo semi-stocastico. La caratteristica fondamentale delle previsioni probabilistiche è quella di considerare l'incertezza associata ai valori previsti, determinando gli intervalli di confidenza delle variabili demografiche e dando la possibilità all'utente di poter scegliere autonomamente il grado di fiducia da assegnare ai risultati.

Rispetto all'approccio "deterministico" usato in passato dall'Istat, dove all'utente non vengono fornite misure di probabilità, si tratta di un avanzamento metodologico significativo. Di fatto, col passaggio all'approccio probabilistico, l'utente cessa di confidare acriticamente sul lavoro dei *projection makers*, che con le varianti "basso/alto" tipiche dell'approccio a scenari deterministici definiscono a priori i confini alternativi alla variante ritenuta "più probabile", generalmente identificata come "scenario centrale".

La quantificazione dell'incertezza in probabilità non rappresenta l'unico vantaggio del modello stocastico sul deterministico. Se ne individua anche un altro che si deve alla più efficace rappresentazione della potenziale evoluzione di una popolazione. Nel modello stocastico, infatti, gli scenari definibili sono infiniti sul piano teorico (per quanto nella realtà, come si vedrà più avanti, se ne selezioni sempre un numero finito), per cui è possibile che ipotesi di bassa sopravvivenza si mescolino con ipotesi di alta fecondità o medio livello delle migrazioni, o il contrario. Invece, le ipotesi degli scenari alto/basso dell'approccio deterministico sono definite perseguendo una logica *output oriented*: lo scenario alto contempla generalmente ipotesi di massimo incremento della sopravvivenza, della fecondità e delle migrazioni, mentre, all'opposto, lo scenario basso contempla solo ipotesi di minimo. La costruzione di tali scenari contrapposti coglie, in effetti, l'obiettivo di determinare un futuro campo di variazione per la popolazione e le sue componenti strutturali, ma si fonda su ipotesi concomitanti che hanno una scarsa possibilità di verificarsi.

Le sezioni successive della nota illustrano in maniera sintetica i passaggi che hanno reso possibile la costruzione delle previsioni e contengono informazioni di carattere generale. Tali sezioni includono informazioni sui seguenti aspetti:

- ✓ popolazione base
- ✓ tecnica di proiezione
- ✓ periodo di previsione
- ✓ panel di esperti
- ✓ questionario degli esperti e modello probabilistico
- ✓ relazione tra le previsioni nazionali e regionali
- ✓ dati di base e componente correttiva di *nowcasting*
- ✓ intervalli di confidenza e scenario mediano
- ✓ previsioni regionali di fecondità

- ✓ previsioni regionali di mortalità
- ✓ previsioni regionali dei movimenti migratori internazionali
- ✓ previsioni regionali delle migrazioni interne
- ✓ confronto con le precedenti previsioni
- ✓ confronto con le previsioni prodotte da Eurostat
- ✓ diffusione dati e termini di utilizzo.

Popolazione base

La popolazione base è quella articolata per sesso, singola classe di età e regione al 1° gennaio 2017, così come identificata dalla rilevazione Istat/Posas. Si tratta della popolazione riconosciuta come ufficiale dall'Istat e allineata alle risultanze del Censimento 2011 della Popolazione. La popolazione include tutte le persone usualmente residenti in Italia, di qualunque cittadinanza, mentre non include né cittadini italiani residenti all'estero, né cittadini illegalmente o irregolarmente presenti sul territorio nazionale che non risultino iscritti presso alcuna anagrafe.

Tecnica di proiezione

Le previsioni sono condotte con tecnica iterativa tra il 1° gennaio e il 31 dicembre di ogni anno, utilizzando il cosiddetto metodo per "coorti-componenti". In corrispondenza di ciascuna classe di età alla popolazione iniziale sono sommate le immigrazioni (dall'estero o da altre regioni) mentre sono sottratti decessi ed emigrazioni (per l'estero o per altre regioni), ottenendo così la popolazione in vita alla fine dell'anno. A ciò vanno aggiunti i nati nel corso dell'anno che, al netto dei decessi e dei movimenti migratori che li riguardano, risultano ancora in vita al 31 dicembre.

Per la popolazione (stock) l'età è definita in anni compiuti al 1° gennaio (da 0 a 110 anni e più). Lo stesso dicasi per i dati di flusso come quelli sui nati, sui morti e sui movimenti migratori. Ciò permette di identificare, sempre e comunque, gli eventi demografici per anno di nascita dei soggetti coinvolti, assicurando la coerenza richiesta all'interno dell'equazione della popolazione.

Si assume che gli eventi demografici possano occorrere linearmente in qualunque momento dell'anno. Tra l'evento di morte e quello di migrazione (per l'interno o con l'estero) si assume che sussista incompatibilità, ossia che non possano coinvolgere un medesimo individuo nello stesso anno.

I decessi si determinano moltiplicando la popolazione residente per classi di età al 1° gennaio per le rispettive probabilità (prospettive) di morte, quelle che cioè interessano soggetti appartenenti alla stessa coorte di nascita.

Le nascite in un dato anno si ottengono in tre passaggi. Nel primo si moltiplica il contingente medio di donne relativo a ogni età feconda (ottenuto come media delle popolazioni di tal età all'inizio e alla fine dell'anno) per il rispettivo tasso di fecondità. Nel secondo si esegue la somma dei nati per età della madre, ottenendo il totale dei nati nell'anno. Nel terzo si scompongono i nati per sesso utilizzando il rapporto fisso di 106 nati di sesso maschile ogni 100 nati di sesso femminile.

Le previsioni hanno un profilo territoriale e sono costruite nella logica del modello multi-regionale, modello che, con particolare riguardo ai movimenti migratori interni, tratta simultaneamente e coerentemente le distinte unità territoriali di riferimento. Il modello parte dalla costruzione di una matrice multi-regionale di probabilità migratorie per regione di origine, regione di destinazione, sesso, ed età. Tale matrice, applicata alla popolazione a rischio di migrare, fornisce, per ogni anno di previsione una serie coerente di immigrati/emigrati da/per ogni regione.

Periodo di previsione

Le previsioni coprono il periodo compreso tra il 1° gennaio 2017 e il 1° gennaio 2066. Lo scopo principale è quello di fornire indicazioni sul futuro sviluppo della popolazione nel breve termine (2025), quindi quello di fornirne nel medio (2045) e lungo termine (2065), precisando che quest'ultime vanno utilizzate con cautela dal momento che i risultati diventano tanto più incerti quanto più ci si allontana dall'anno base (2017). Tale rischio è tanto più concreto quanto più si cala l'attenzione sulle unità territoriali più piccole, come nel caso di alcune regioni italiane.

Panel di esperti

Un panel di esperti nazionali ha supportato l'Istat nella definizione delle ipotesi demografiche relative all'Italia. Le ipotesi relative alle regioni, invece, sono state curate dall'Istat sulla base di un'apposita metodologia "ponte" tra le ipotesi nazionali e quelle regionali. Gli esperti che hanno risposto al questionario, fornendo informazioni utili e complete a definire le ipotesi, sono stati 24 e precisamente:

- ✓ Elena Ambrosetti – Università degli studi Sapienza di Roma
- ✓ Bruno Arpino – Universitat Pompeu Fabra di Barcellona (Spagna)
- ✓ Nicola Barban – University of Oxford (Regno Unito)
- ✓ Elisabetta Barbi – Università degli studi Sapienza di Roma
- ✓ Patrizia Benassi – Ufficio di Statistica della Provincia di Modena
- ✓ Gian Carlo Blangiardo – Università degli studi di Milano Bicocca
- ✓ Giovanna Boccuzzo – Università degli studi di Padova
- ✓ Corrado Bonifazi – Istituto di ricerche sulla popolazione e le politiche sociali
- ✓ Annalisa Busetta – Università degli studi di Palermo
- ✓ Oliviero Casacchia – Università degli studi Sapienza di Roma
- ✓ Alessandra De Rose – Università degli studi Sapienza di Roma
- ✓ Viviana Egidi – Università degli studi Sapienza di Roma
- ✓ Patrizia Farina – Università degli studi di Milano Bicocca
- ✓ Giuseppe Gabrielli – Università degli studi di Napoli Federico II
- ✓ Frank Heins – Istituto di ricerche sulla popolazione e le politiche sociali
- ✓ Roberto Impicciatore – Alma Mater Studiorum Università di Bologna
- ✓ Maria Cristina Migliore – Istituto di ricerche economiche e sociali del Piemonte
- ✓ Eros Moretti – Università Politecnica delle Marche
- ✓ Alessandro Rosina – Università Cattolica del Sacro Cuore di Milano
- ✓ Fiorenzo Rossi – Università degli studi di Padova
- ✓ Salvatore Strozza – Università degli studi di Napoli Federico II
- ✓ Laura Terzera – Università degli studi di Milano Bicocca
- ✓ Maria Rita Testa – Vienna Institute of Demography (Austria)
- ✓ Cecilia Tomassini – Università degli studi del Molise

In tutte le fasi che hanno riguardato la costruzione dell'impianto metodologico alla base delle previsioni, l'Istat si è avvalsa della concreta cooperazione di ricercatori esterni e, in particolare, di:

- ✓ Francesco Billari – University of Oxford (Regno Unito)
- ✓ Rebecca Graziani – Università Bocconi di Milano
- ✓ Eugenio Melilli – Università Bocconi di Milano

Questionario degli esperti e modello probabilistico

Il metodo probabilistico adottato si basa sulle opinioni degli esperti (*expert-based model*) circa l'evoluzione futura dei più importanti indicatori demografici e rientra nella classe più ampia dei modelli *random scenario*. Tale modello, utilizzato per la definizione degli scenari probabilistici a livello nazionale, si fonda sull'elicitazione di una serie di parametri da cui viene derivata la futura evoluzione stocastica di ciascuna componente demografica. Gli esperti sono chiamati a fornire dei valori a un dato anno "t" riguardo a una serie di indicatori demografici di sintesi, condizionatamente ai valori assunti dagli stessi indicatori in istanti di tempo precedenti l'anno "t" (Billari, Graziani e Melilli, 2012).

Il metodo stocastico utilizzato ha il vantaggio di essere semplice e flessibile. Nel questionario, infatti, le componenti demografiche necessarie sono sintetizzate attraverso i seguenti indicatori: il numero medio di figli per donna; la speranza di vita alla nascita distinta per sesso; le immigrazioni e le emigrazioni con l'estero. Le altre informazioni necessarie alla produzione delle previsioni, come quelle riguardo alla distribuzione per età (cadenza) degli eventi demografici, sono tenute appositamente fuori e lavorate in seguito al fine di rendere parsimonioso il questionario e il modello previsivo medesimo.

Per ogni indicatore demografico si prendono in considerazione due istanti temporali: un anno intermedio "t1" e un anno "t2" corrispondente all'ultimo anno di previsione. Nel questionario sottoposto agli esperti si considera "t0 = 2015", "t1 = 2040", "t2 = 2065", generando in questo modo due sotto-intervalli, 2015-2040 e 2040-2065. Indicare il valore della speranza di vita alla

nascita nell'anno 2065, dato il valore previsto nel 2040, è un pratico esempio di come funziona il meccanismo.

PROSPETTO A1. VALORI MEDI, VARIANZE E CORRELAZIONI OTTENUTE SOTTO LE IPOTESI DEGLI ESPERTI PER TIPO DI INDICATORE. Italia, anni 2015, 2040 e 2065.

Indicatore	Numero medio di figli per donna	Speranza di vita alla nascita – Uomini	Speranza di vita alla nascita – Donne	Immigrazioni dall'estero (migliaia)	Emigrazioni per l'estero (migliaia)
Anno 2015					
Livello osservato	1,35	80,1	84,6	280	147
Anno 2040					
Ipotesi media	1,51	83,7	88,0	283	124
Ipotesi alta	1,71	85,0	89,7	378	185
Varianza	0,024	0,970	1,662	5.470	2.272
Anno 2065					
Ipotesi media condizionata all'ipotesi media del 2040	1,59	86,0	90,3	271	127
Ipotesi media condizionata all'ipotesi alta del 2040	1,82	87,3	91,8	347	185
Ipotesi alta condizionata all'ipotesi media del 2040	1,75	87,2	91,5	352	188
Varianza	0,046	1,539	2,272	7.523	4.418
Correlazione anni 2040-2065 (min 0, max 1)					
Coefficiente di correlazione	0,68	0,54	0,59	0,47	0,47

FIGURA A1. EVOLUZIONE DEL NUMERO MEDIO DI FIGLI PER DONNA SULLA BASE DI 3.000 SIMULAZIONI STOCASTICHE OTTENUTE A PARTIRE DALLE VALUTAZIONI DEGLI ESPERTI. Italia, anni 2016- 2065.

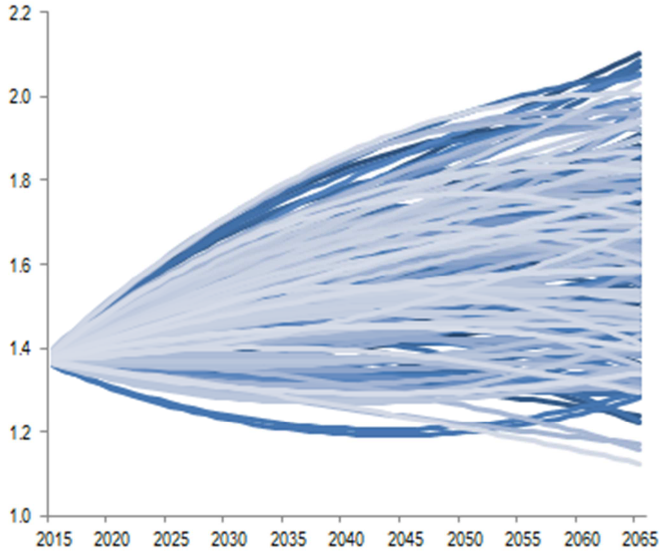

Gli indicatori demografici sono assunti, per semplicità di ipotesi, tra loro indipendenti (ad esempio, la propensione media di fecondità espressa dalle donne non è influenzata dal livello delle migrazioni e viceversa), benché il modello proposto permetta nella sua versione generalizzata la possibilità di far interagire tra loro le componenti demografiche in gioco. Si assume, inoltre, che la coppia di elicitazioni al 2040 e al 2065 di un dato indicatore possegga una distribuzione normale bivariata.

Sotto tali condizioni, una volta ottenuti i valori medi di risposta forniti dagli esperti è possibile stimare la varianza associata in ciascuno dei due istanti temporali nonché la correlazione tra il primo e il secondo (Prospetto A1). Sulla base delle corrispondenti distribuzioni normali bivariate sono state quindi effettuate 3000 simulazioni col metodo *Markov Chain Monte Carlo*.

L'ultimo passo di stima è finalizzato al calcolo dei valori di ciascun parametro negli anni intermedi i due intervalli 2015-2040 e 2040-2065. Tale attività è realizzata, per ciascuna delle 3000 simulazioni, mediante interpolazione con curve di tipo quadratico, passanti per i punti noti in corrispondenza degli anni 2015, 2040 e 2065. Si è così pervenuto alla definizione di 3000 curve stocastiche per ogni indicatore demografico considerato a livello nazionale. A titolo di esempio, la Figura A1 descrive il fascio di curve relativo al numero di figli per donna, ricavato dalla procedura sopra descritta.

La scelta di considerare un numero di 3000 simulazioni è frutto di un compromesso tra due necessità, entrambe strategiche: quella di rappresentare fedelmente l'aleatorietà degli eventi demografici e quella di ottimizzare i tempi macchina di elaborazione delle previsioni. Questi ultimi, nonostante l'odierna disponibilità di strumenti hardware/software sempre più potenti e sofisticati, costituiscono a loro volta un aspetto tecnico tutt'altro che secondario considerando la notevole mole di dati trattati.

Relazione tra le previsioni nazionali e regionali

Il modello probabilistico fornisce un set di 3000 simulazioni nazionali per ciascun indicatore demografico di sintesi. Poiché l'obiettivo delle previsioni Istat è anche quello di fornire indicazioni a livello territoriale, proseguendo la tradizione del modello multiregionale, è stata implementata una procedura "ponte" tra la definizione degli input nazionali e quelli regionali. L'approccio perseguito è dunque di natura *top-down* dal lato della costruzione delle ipotesi mentre, come si vedrà più avanti, è di natura *bottom-up* dal lato della produzione degli output finali.

L'azione principale è quella di derivare 3000 scenari regionali stocastici dai 3000 scenari nazionali frutto della consultazione tra gli esperti. La prima operazione in tal senso è quella di elaborare una previsione deterministica intermedia, applicando il modello multi-regionale a coorti componenti e includendo la modellazione delle migrazioni interregionali. Da tale previsione, ottenuta estrapolando le tendenze regionali ritenute più probabili per ciascuna componente (cfr. paragrafi successivi), vengono ricavati gli stessi indicatori di sintesi oggetto del modello stocastico precedentemente descritto, ossia numero medio di figli per donna, speranza di vita alla nascita maschile e femminile, movimenti migratori con l'estero. Questa prima previsione intermedia, unica e deterministica, somiglia in sostanza a quella che nelle passate edizioni delle previsioni veniva contraddistinta col termine di "scenario centrale".

A questo punto, la transizione dal modello regionale deterministico al modello regionale stocastico si realizza moltiplicando, e reiterando 3000 volte il procedimento, la previsione dell'indicatore di sintesi deterministico regionale per il rapporto intercorrente tra la previsione nazionale stocastica e quella deterministica. In formula, indicando con "n" la generica simulazione (n=1, … , 3000), con "j" il codice territoriale regionale, con DR la previsione regionale deterministica, con SR quella stocastica, con DN e SN, rispettivamente, le previsioni nazionali deterministiche e stocastiche, si ha:

$$ {}_{n}SR_{t}^{j} = DR_{t}^{j} \times \frac{{}_{n}SN_{t}}{DN_{t}} $$

agganciando così, a ogni simulazione, il vettore dei valori regionali al valore nazionale stocastico di riferimento. Si noti che per quanto concerne gli indicatori sintetici delle immigrazioni e delle emigrazioni con l'estero si ha che:

$$ DN_{t} = \sum_{j} DR_{t}^{j} \text{ e } {}_{n}SN_{t} = \sum_{j} {}_{n}SR_{t}^{j} \,. $$

Ottenuti gli indicatori sintetici stocastici a livello regionale, si passa alla costruzione degli input necessari all'applicazione del metodo per coorti-componenti, ossia le probabilità prospettive di morte per sesso ed età, i tassi specifici di fecondità per età della madre e la distribuzione degli immigrati/emigrati con l'estero per sesso ed età. La procedura associa quindi a ogni indicatore di sintesi la relativa cadenza del fenomeno (sesso ed età). Quest'ultima, non trattata in via stocastica, è quella che deriva dal modello deterministico regionale e, di simulazione in simulazione, riadattata allo specifico indicatore sintetico stocastico.

L'abbinamento dei 3000 vettori di probabilità di morte (ciascun vettore sviluppa un numero di elementi pari a "numero di regioni X classi di età X sesso X anni di previsione") con i 3000 vettori

di fecondità, e gli altrettanti sulle immigrazioni e le emigrazioni con l'estero e, infine, con le 3000 matrici O/D di probabilità di migrazione interna, è di tipo casuale.

Dopo aver introdotto una componente correttiva di *nowcasting* (cfr. paragrafo successivo) relativa ai primissimi anni di previsione, il modello coorti componenti viene fatto a quel punto girare 3000 volte, ottenendo così gli output richiesti: popolazione per sesso ed età, flussi demografici per sesso ed età, più la serie di indicatori demografici di supporto all'analisi che va dai tassi generici (di natalità, di mortalità ecc..) agli indicatori strutturali (età media, indice di dipendenza, di vecchiaia ecc..).

I risultati a livello nazionale (nonché di ripartizione geografica) nell'ambito di ciascuna simulazione regionale si ottengono per somma (approccio *bottom-up*). Pertanto, l'ammontare della popolazione prevista, dei decessi, e delle migrazioni, classificate per sesso ed età, e delle nascite per età della madre che si determinano a livello nazionale (o di ripartizione) sono la sommatoria delle traiettorie previsive regionali. Anche gli assunti livelli nazionali (e di ripartizione) relativi agli indicatori di sintesi posti in diffusione, riguardo ad esempio la speranza di vita o il numero medio di figli per donna, sono ricalcolati ex-post sulla base di tali riepiloghi regionali.

E' opportuno segnalare che la stocasticità introdotta a livello regionale, mutuata *top-down* da quella nazionale e limitata ai soli indicatori di sintesi, potrebbe risultare non sempre sufficiente a riprodurre l'aleatorietà dei vari eventi demografici. Ciò è particolarmente vero nelle piccole realtà territoriali, dove l'incertezza tende a essere relativamente maggiore. Per questa ragione è più opportuno parlare di approccio semi-stocastico quando ci si riferisce alle previsioni regionali, per quanto la numerosità delle simulazioni condotte offra comunque ampia garanzia di rappresentatività della variabilità su scala regionale.

Una seconda osservazione riguarda il fatto che nel modello Istat è esclusa una trattazione statistica generalizzata della covarianza tra le regioni (ad esempio: la previsione di incremento/decremento della fecondità in una data regione quanto condiziona o quanto è a sua volta condizionata dalla previsione di incremento/decremento in un'altra). A questa strada, esclusa anche per ragioni di parsimonia del modello statistico, se ne è preferita un'altra, quella della convergenza territoriale. Infatti, il modello regionale deterministico iniziale, successivamente trasformato in modello stocastico attraverso la procedura sopra descritta, è costruito su ipotesi di convergenza a lunghissimo termine (2115, ben oltre l'ultimo anno delle previsioni) tra le regioni per ciascuna componente demografica fondamentale. Ciò comporta che i 3000 scenari stocastici regionali raffigurano 3000 diverse ipotesi di convergenza dei comportamenti demografici sul territorio.

L'ipotesi principale alla base della convergenza è che le differenze socio-economiche e culturali attualmente in essere tra le regioni costituenti il territorio italiano siano destinate a scomparire nel lungo termine. Perciò, il loro progressivo annullamento comporterebbe anche un generalizzato riavvicinamento dei comportamenti demografici. L'idea di convergenza non è nuova in demografia ed esistono molti esempi di previsioni demografiche che la seguono (Eurostat e ONU, in particolare), tra cui anche quelle passate dell'Istat. Nelle previsioni Istat la convergenza è intesa come spostamento progressivo di un dato comportamento demografico verso un punto molto distante nel futuro che rappresenta l'istante di piena convergenza regionale (nel senso che a quel punto i valori risulterebbero identici per le diverse regioni), ma che in realtà è lungi dall'essere raggiunto all'interno dell'orizzonte previsivo considerato (2017-2065). Di fatto, risulta corretto in tale circostanza parlare più di modello di semi-convergenza che di modello di convergenza piena.

Dati di base e componente correttiva di nowcasting

Le ipotesi definite a livello regionale nel modello deterministico preliminare, prima del passaggio al modello stocastico, sono state ottenute estrapolando i futuri trend dall'analisi delle serie storiche osservate. In particolare tali ipotesi sono state definite ricorrendo alle seguenti serie di dati:

- ✓ per la fecondità, i tassi specifici per età della madre del periodo 1977-2014;
- ✓ per la mortalità, le probabilità di morte per sesso ed età del periodo 1974-2014;
- ✓ per le migrazioni interne e internazionali, i trasferimenti di residenza per sesso ed età del 2010-2014.

Prima di essere lanciate a pieno regime lungo l'orizzonte previsivo col metodo coorti-componenti, le previsioni incorporano un fattore correttivo di *nowcasting* (dal termine *nowcast*=previsione del presente, contrapposto al più noto termine *forecast*=previsione del futuro). Con tale operazione si intende fare in modo che la previsione relativa ai primissimi anni risulti quanto più in linea alla tendenza emersa nell'ultimo periodo storico, pur preservando quella che è la potenziale variabilità

del fenomeno studiato. Si tende cioè a evitare che, per il semplice fatto che mancano all'osservazione statistica uno o più anni antecedenti l'anno base delle previsioni, l'esecuzione di quest'ultime comporti in avvio una distorsione dovuta alla creazione di uno scalino. Queste previsioni incorporano quali nowcast quelle rilasciate dall'Istat nel comunicato "Indicatori demografici - Stime per l'anno 2017" dell'8 febbraio 2018.

Sul piano operativo la rivisitazione delle ipotesi di breve termine si realizza applicando dei fattori di correzione. Sia, ad esempio, E_b il numero di eventi demografici previsti nel primo anno sulla base dello scenario mediano. E sia, invece, $\hat{E}_b$ il valore osservato di tali eventi oppure, in assenza del valore realmente osservato, la migliore stima che si possa ottenere (ad esempio, utilizzando procedure di *nowcasting* o analoghi modelli statistici). Il rapporto

$$r_b = \frac{\hat{E}_b}{E_b}$$

rappresenta il fattore di correzione da applicare alle misure statistiche che danno luogo agli eventi di tipo "E" nell'anno "b". Se ad esempio tali eventi fossero il numero totale di nascite allora la quantità

$$\hat{f}^s_{x,b} = r_b \cdot f^s_{x,b} \qquad x = 14,...,50 \qquad s = 1,...,3000$$

rappresenta la serie dei tassi specifici di fecondità per età della madre (s-sima simulazione) corretta per l'anno "b". Analoghe considerazioni valgono per la determinazione dei coefficienti di correzione inerenti la mortalità e i movimenti migratori.

I fattori di correzione vengono applicati per un periodo limitato dell'orizzonte previsivo e determinando pesi correttivi che tendono progressivamente a uno. In particolare, il numero di anni per cui il fattore di correzione si applica alla serie di interesse si desume da:

$$Y = abs(1 - r_b) \cdot \varepsilon$$

con ε quantità arbitraria, mentre i livelli dei fattori di correzione per gli anni successivi a "b", per un totale di "Y" anni, sono dati da:

$$r_t = \frac{r_b \cdot (b + Y - t) + (t - b)}{Y} \qquad t = b, b+1,..., b+Y-1$$

Intervalli di confidenza e scenario mediano

Una volta lanciata la procedura di calcolo inerente le 3000 simulazioni regionali, il margine di incertezza è calcolato per tutti i possibili livelli informativi, dalla popolazione prevista ai dati di flusso, sulla base delle componenti strutturali del sesso e dell'età. Tali margini di incertezza dipendono a loro volta dall'incertezza insita nei futuri livelli di mortalità, fecondità e migratorietà che sono anch'essi resi disponibili. La diffusione dei risultati contempla il rilascio dei soli intervalli di confidenza del 90%, dell'80% e del 50% ma è possibile definire intervalli su qualunque scala di interesse. L'intervallo di confidenza restituisce l'informazione su quanto sia probabile che un determinato indicatore demografico ricada entro prefissati limiti. Sotto questo punto di vista è opportuno ricordare che tale probabilità rappresenta essa stessa una previsione, in quanto fondata su ipotesi la cui validità è incerta. In nessun caso, inoltre, gli estremi dell'intervallo di confidenza vanno interpretati come estremi limiti, superiori o inferiori, del futuro comportamento demografico.

La costruzione di un intervallo di confidenza è qui fondata sulla determinazione dei percentili nella distribuzione delle 3000 simulazioni. Ad esempio, l'intervallo di confidenza al 90% per un dato indicatore è determinato considerando i valori della distribuzione che ricadono tra il 5° e il 95° percentile. Si ricorda, inoltre, che l'incertezza si riferisce sempre e comunque al dominio dello specifico parametro stimato. I limiti dell'intervallo di confidenza per un dato livello gerarchico sono stimati in proprio, e non costruiti per sommatoria di limiti ottenuti a un livello di disaggregazione gerarchicamente inferiore. Il criterio trova applicazione anche in contesti gerarchici non territoriali; ad esempio nella composizione per età della popolazione o in quella per sesso.

Con lo scopo di definire una previsione "puntuale" che possa essere adottata quale riferimento più probabile dell'evoluzione demografica futura è stato definito lo "scenario mediano". Tale scenario corrisponde a una 3001-esima simulazione, ottenuta per costruzione, ma che di fatto non è stata

rilevata nel campo di osservazione delle 3000 simulazioni. Il set di ipotesi viene identificato prendendo a riferimento il valore mediano tra tutte le simulazioni a livello delle singole componenti demografiche (fecondità, mortalità, migrazioni) nell'ambito delle possibili combinazioni delle covariate età, regione e anno di previsione. Ad esempio, il tasso specifico di fecondità all'età di 30 anni della regione Lombardia, dell'anno 2045, dello scenario mediano è identificato come il valore mediano con tali caratteristiche individuato tra tutte le simulazioni. Il medesimo tasso specifico ma all'età successiva, o nell'anno successivo, è individuato con lo stesso procedimento ma esso scaturisce, verosimilmente, da una simulazione diversa. Per l'identificazione dello scenario mediano sulla mortalità e sulle migrazioni la procedura è identica ma con l'ulteriore covariata del sesso. Per le migrazioni interne, inoltre, le covariate territoriali riguardano la regione di origine e di destinazione.

Lo scenario è pertanto "mediano" dal lato degli input fondamentali. Dal punto di vista degli output (popolazione e flussi previsti) che tale scenario genera una volta lanciata la procedura per coorti-componenti, per le proprietà tipiche della mediana esso restituisce valori molto prossimi a quelli mediani.

Previsioni regionali di fecondità

Per la fecondità regionale le previsioni hanno riguardato i classici parametri di intensità e cadenza, ossia il numero medio di figli per donna e la distribuzione dei tassi specifici di fecondità per età della madre.

Il numero medio di figli per donna è stato rappresentato ricorrendo a modelli di tipo ARIMA(n,p,k) ricercando, distintamente per singola regione, quello più idoneo a prevedere l'intensità futura del comportamento riproduttivo, sulla base della serie storica 1977-2014. I modelli prevalentemente utilizzati sono quelli che rientrano nelle categorie (1,1,0) e (2,1,0), con o senza intercetta.

Il profilo per età della fecondità è stato modellato ricorrendo a un sistema di funzioni *quadratic splines* (Schmertmann, 2003). Tale modello descrive funzionalmente la curva dei tassi specifici di fecondità standardizzati in funzione di tre parametri: l'età di inizio dell'età fertile α; l'età P in cui la fecondità raggiunge il suo livello massimo; l'età H, successiva a P, nella quale la fecondità si dimezza rispetto al livello massimo. Per tasso specifico di fecondità standardizzato si intende il tasso specifico di fecondità normalizzato all'unità in corrispondenza del valore massimo individuato all'interno della sua distribuzione per età.

Il modello di *quadratic splines* adatta cinque polinomi di secondo grado alle curve di fecondità. La funzione finale risulta continua con la derivata prima anch'essa continua. Inoltre, grazie ad opportune restrizioni matematiche essa è univocamente determinata dai tre parametri [α, P, H] sopra menzionati.

In pratica, la previsione del tasso specifico di fecondità si trasforma nella previsione dei tre parametri che lo esprimono funzionalmente, una volta stimatane la serie nel periodo 1977-2014. Per fare questo è stata adottata un'ipotesi di convergenza tra le regioni italiane, assumendo che le differenze territoriali in termini di comportamento demografico tendano a diminuire nel lungo periodo. Dal punto di vista operativo la piena convergenza tra le regioni è stata fissata nel 2115. In particolare il vincolo di convergenza prevede che, dal 2017 al 2115, i parametri del vettore regionale [α, P, H] convergano linearmente ai valori di un ipotetico vettore nazionale, appositamente disegnato per l'operazione.

Previsioni regionali di mortalità

Le previsioni di mortalità sono state prodotte ricorrendo al modello di Lee-Carter (1992) nella variante proposta da Lee-Miller (2001), modello nel quale la procedura di *adjustment* riconduce le probabilità teoriche di morte a riprodurre precisamente il livello osservato della speranza di vita alla nascita, anziché il totale dei decessi osservati come nella versione originale. Inoltre, il modello è applicato alla distribuzione per età delle probabilità di morte anziché a quella dei tassi specifici di mortalità della formulazione originale.

Il modello approssima la forma logaritmica delle probabilità di morte utilizzando tre parametri sintetici, di cui uno legato al trend [k(t)] e due legati alla distribuzione per età [(a(x), b(x)].

Come per la fecondità, anche per la mortalità la costruzione del modello origina dalla definizione di uno scenario di riferimento provvisorio a livello nazionale. La previsione si determina proiettando nel futuro il solo parametro nazionale di trend k(t), la cui serie è individuata sul periodo 1974-2014,

mentre i parametri a(x) e b(x) rimangono in questa fase invarianti nel tempo. In particolare, per via della sostanziale linearità il parametro k(t) è stato proiettato al 2065 con la tecnica del *random walk with drift*.

Le ipotesi a livello regionale vengono fatte discendere dal provvisorio scenario di riferimento nazionale, stimando in primo luogo i valori regionali dei tre parametri nel 1974-2014 con la stessa metodologia e, successivamente, facendo convergere ogni parametro regionale al corrispondente parametro nazionale al 2115. Pertanto, come conseguenza del processo di convergenza e diversamente dall'impostazione classica del modello di Lee-Carter, qui si fanno variare nel tempo anche i parametri regionali a(x) e b(x).

Previsioni regionali dei movimenti migratori internazionali

Al fine di catturare le tendenze più recenti, le previsioni dei flussi migratori con l'estero concentrano l'analisi solo sugli ultimi cinque anni, vale a dire sul 2010-2014. Questa necessità, considerando la complessità di prevedere flussi migratori internazionali ricorrendo ad analisi di serie storiche, porta all'uso di un modello molto semplificato. Senza dimenticare che a questo livello delle operazioni si tratta di strutturare un modello deterministico intermedio, i cui valori sono in seguito calibrati sulle intensità prodotte dal modello stocastico *expert-based*.

Nel primo anno di proiezione i valori totali delle immigrazioni e delle emigrazioni con l'estero sono posti pari al valore medio osservato nel corso degli ultimi cinque anni. In conformità con il quadro generale di convergenza del modello deterministico, si suppone quindi che in ciascuna regione ingressi e uscite convergano linearmente nel lungo periodo (2115) al medesimo livello, ossia alla semisomma iniziale dei due valori.

Una volta determinati i totali dei flussi in ingresso e in uscita fino al 2065, le distribuzioni per sesso ed età associate vengono derivate applicando il modello Castro-Rogers (Rogers and Castro, 1981) alla serie 2010-2014. Con tale modello si dimostra che il caratteristico profilo per età delle migrazioni (qualunque esse siano, in ingresso o in uscita, con l'estero o con l'interno) può essere descritto, indipendentemente dall'intensità del fenomeno, da una funzione matematica composta di quattro componenti additive e fino a 11 parametri predittivi. Tali parametri, la cui stima nel periodo osservato è prodotta grazie a una procedura generalizzata per modelli non lineari (categoria nella quale la funzione Castro-Rogers ricade in pieno), sono tenuti costanti nel periodo di previsione. Il risultato conclusivo è, dunque, che l'intensità globale dei flussi migratori con l'estero possa variare nel tempo ma sulla base di una composizione per età fissa.

Previsioni regionali delle migrazioni interne

Le migrazioni interregionali sono trattate secondo un approccio multidimensionale, che permette di considerare simultaneamente le aree di origine e destinazione dei flussi migratori, e di definire gli ingressi in una determinata area come somma delle uscite con quella destinazione da tutte le altre aree del sistema. Il sistema è per costruzione coerente per tutti gli anni di previsione in quanto la riga e la colonna marginali della matrice O/D, corrispondenti rispettivamente ai flussi in entrata e in uscita in/da ciascuna regione, danno la stessa somma, corrispondente all'ammontare complessivo dei movimenti interni al territorio nazionale.

La probabilità di migrazione specifica per età (110), sesso (2), regione di origine (21) e di destinazione (21) costituisce la componente elementare della matrice O/D composta di 110x2x21x21=97020 celle per ciascun anno di calendario. Le probabilità sono stimate sulla base dei livelli osservati nelle singole annualità del periodo 2010-2014. I vettori di probabilità così ottenuti, a livello di ciascuna annualità, sono successivamente perequati utilizzando la funzione Castro-Rogers. Pertanto, indicando con

$$m^{ij}_{x,s,t}$$

la generica probabilità prospettiva di migrare per un individuo di età "x" e sesso "s" tra la regione "i" e la regione "j" relativa all'annualità "t" (t=2010, … , 2014), si assume che questa rappresenti una variabile casuale di tipo normale con media pari al valore medio del quinquennio e varianza pari alla varianza rilevata nel quinquennio:

$$\mu^{ij}_{x,s} = E\left(m^{ij}_{x,s,t}\right)$$

$$\sigma^{ij}_{x,s} = E\left[\left(m^{ij}_{x,s,t} - \mu^{ij}_{x,s}\right)^2\right].$$

Dalle suddette variabili casuali vengono estratti casualmente 3000 valori per ciascuno dei 97020 elementi della matrice di O/D, dando così luogo alla creazione casuale di 3000 matrici tra loro diverse. La matrice O/D relativa allo scenario stocastico mediano è identificata prendendo a riferimento il valore mediano tra tutte le simulazioni nell'ambito delle possibili combinazioni delle covariate sesso, età, regione di origine e destinazione. Tale matrice mediana è quella anche usata con lo scopo preliminare di produrre la previsione deterministica della popolazione, antecedente la transizione al modello stocastico vero e proprio (cfr. precedente paragrafo sulla relazione tra previsioni nazionali e regionali).

Si noti che nell'ambito di ciascuna simulazione (compresa quella relativa allo scenario mediano) la matrice O/D è supposta invariante nel tempo. L'ipotesi alla base del modello si fonda, infatti, sul mantenimento per tutto il periodo previsivo di una propensione alla mobilità che rimanga costante. Ciò comporta che i flussi migratori interni evolvano nel tempo solo in virtù delle variazioni che interessano livello e struttura per età della popolazione esposta al rischio di migrare.

Confronto con le precedenti previsioni

Rispetto alle precedenti previsioni demografiche, in base 1° gennaio 2016, sono stati apportati due cambiamenti: 1) l'aggiornamento della base di popolazione per sesso, età e regione al 1° gennaio 2017; 2) la parziale rivisitazione nel breve termine delle ipotesi demografiche nei confronti dei principali indicatori di sintesi. Il resto dell'impianto metodologico è identico a quello utilizzato in occasione del precedente round previsivo. Il prospetto A2 evidenzia come il processo di rivisitazione delle ipotesi sulla fecondità, sui movimenti migratori con l'estero e sulla speranza di vita abbia riguardato quasi soltanto i primi anni di previsione. Già nel medio termine (2045) e ancora più nel lungo termine, le ipotesi sottostanti lo scenario mediano 2017 ricalcano quelle del round previsivo precedente.

PROSPETTO A2. CONFRONTO DELLE IPOTESI PER I PRINCIPALI INDICATORI SINTETICI TRA GLI SCENARI MEDIANO IN BASE 2016 E 2017. Italia, anni 2017, 2025, 2045 e 2065.

Scenario	Numero medio di figli per donna	Speranza di vita alla nascita – Uomini	Speranza di vita alla nascita – Donne	Immigrazioni dall'estero (migliaia)	Emigrazioni per l'estero (migliaia)
Anno 2017					
Mediano 2016	1,36	80,9	85,5	294	155
Mediano 2017	1,34	80,6	85,0	337	153
Anno 2025					
Mediano 2016	1,44	81,9	86,3	298	141
Mediano 2017	1,44	81,9	86,3	324	140
Anno 2045					
Mediano 2016	1,53	84,3	88,5	286	129
Mediano 2017	1,53	84,3	88,5	288	129
Anno 2065					
Mediano 2016	1,59	86,1	90,2	271	132
Mediano 2017	1,59	86,1	90,2	271	132

Alla luce delle ridotte modifiche introdotte, in termini di ipotesi demografiche di base, anche i risultati prodotti non presentano rilevanti differenze tra i due ultimi successivi round di previsioni. In particolare, sotto la lente dell'interpretazione delle future dinamiche demografiche nulla cambia in termini di riduzione della popolazione residente nel lungo termine, così come in termini del suo progressivo invecchiamento.

Dal lato dei flussi previsti nel tratto di comune proiezione (2017-2065) si intravede una dinamica solo lievemente più marcata nelle previsioni in base 2017. Ciò dipende in particolare dalla più importante rivisitazione delle ipotesi relative agli immigrati dall'estero, pari a 14,6 milioni, oltre 530 mila individui in più rispetto alle previsioni in base 2016. Poco significativo, invece, il confronto per le altre componenti di bilancio tra i due successivi round previsivi. Sempre prendendo a riferimento

la cumulata del tratto storico 2017-2065, nelle previsioni in base 2017 si rilevano 27 mila nascite in più, 79 mila decessi in più e 18 mila emigrazioni per l'estero in meno. Come conseguenza di tali differenti dinamiche la popolazione prevista al 1° gennaio 2065 nello scenario mediano in base 2017 ammonta a 54,1 milioni, ossia oltre 400 mila unità in più rispetto a quella che alla medesima data si prevedeva essere la popolazione nello scenario mediano in base 2016.

Anche l'aggiornamento della popolazione base delle previsioni non ha comportato rilevanti cambiamenti. Lo scenario mediano in base 2016 prevedeva una popolazione residente al 1° gennaio 2017 di 60 milioni 667 mila unità mentre la popolazione ufficiale alla medesima data conta 60 milioni 589 mila unità, quindi una differenza tra dato previsto e dato rilevato di 77 mila unità. Tenendo inoltre conto del fatto che, nel corso del 2016, si sono registrate operazioni di revisione e aggiustamento delle anagrafi comunali che hanno determinato un saldo negativo di 59 mila unità, e sottolineando che tali operazioni amministrative non rientrano nella sfera del modello di previsione, la differenza "reale" (al netto delle revisioni anagrafiche) tra la popolazione prevista nello scenario mediano 2016 e la popolazione ufficialmente rilevata in seguito è pari a circa 18 mila unità.

Confronto con le previsioni realizzate da Eurostat

Al fine di confrontare le previsioni prodotte dall'Istat con quelle di altri Enti ha senso prendere a riferimento le previsioni rilasciate il 24 febbraio 2017 da Eurostat (le ultime disponibili), l'istituto statistico dell'Unione europea che assolve il compito di produrle con regolare cadenza per tutti i Paesi membri.

PROSPETTO A3. CONFRONTO TRA LE IPOTESI SUI PRINCIPALI INDICATORI SINTETICI DELLO SCENARIO MEDIANO IN BASE 2017 E DELLO SCENARIO EUROSTAT IN BASE 2015. Italia, anni 2017, 2025, 2045 e 2065.

Scenario	Numero medio di figli per donna	Speranza di vita alla nascita – Uomini	Speranza di vita alla nascita – Donne	Saldo migratorio con l'estero (migliaia)
Anno 2017				
Istat 2017 mediano	1,34	80,6	85,0	184
Eurostat 2015	1,33	80,8	85,5	145
Anno 2025				
Istat 2017 mediano	1,44	81,9	86,3	183
Eurostat 2015	1,39	81,9	86,4	182
Anno 2045				
Istat 2017 mediano	1,53	84,3	88,5	159
Eurostat 2015	1,51	84,3	88,5	204
Anno 2065				
Istat 2017 mediano	1,59	86,1	90,2	139
Eurostat 2015	1,63	86,4	90,4	172

Occorre sottolineare in premessa che, nonostante l'estrema comparabilità sul piano della tecnica proiettiva e della freschezza informativa, i due esercizi presentano alcune differenze metodologiche. Tra queste, in primo luogo, il fatto che le previsioni di Eurostat hanno come base il 1° gennaio 2015 e proiettano la popolazione al 2080. In secondo luogo, va menzionata la diversità dei modelli. Quello Eurostat è di tipo uni-nazionale, ossia proietta la popolazione residente in Italia nel suo insieme non tenendo conto dello sviluppo delle regioni. Inoltre, l'approccio implementato da Eurostat è di tipo deterministico, sviluppa cioè un solo scenario e senza varianti alternative che tengano in qualche modo conto dell'incertezza.

Il prospetto A3 presenta le principali ipotesi di scenario messe a confronto. Per quanto attiene i flussi migratori, il confronto è limitato al saldo migratorio con l'estero in quanto Eurostat costruisce le ipotesi direttamente su tale indicatore (la distinzione tra immigrati ed emigrati è ottenuta in seguito, fissando i secondi pari alla media osservata negli ultimi cinque anni e ottenendo i primi per differenza).

Le ipotesi sulla fecondità sono abbastanza simili, con l'unica differenza sostanziale che nelle previsioni Istat si assume un recupero della fecondità più accentuato nei primi anni di previsione.

Le ipotesi sulla sopravvivenza sono anch'esse molto ravvicinate, benché quelle di Eurostat risultino più ottimistiche nel lungo termine. Un quadro maggiormente differenziato tra i due Istituti è quello che si prospetta nei confronti dei flussi migratori. Nelle previsioni Istat il saldo migratorio con l'estero è inizialmente più alto di quello previsto da Eurostat, quindi dopo il 2025 è in quest'ultimo modello dove si fanno più favorevoli le condizioni per una pressione migratoria più intensa. Tale evidenza si deve in parte alla metodologia di Eurostat, la quale, oltre a prevedere l'evoluzione di fondo delle migrazioni nette, incorpora nel modello una componente additiva di *replacement-migration*. Tale componente assegna annualmente una quota aggiuntiva di migranti netti nella misura pari al 10% della riduzione che si riscontra nella popolazione in età attiva (15-64 anni).

Lo sviluppo delle diverse ipotesi demografiche da luogo a delle differenze in termini di risultati attesi che, per quanto riguarda l'evoluzione della popolazione totale, è possibile apprezzare dalla figura A2. Nei primissimi anni Eurostat prevede una popolazione più alta. Si tratta di un effetto dovuto alla differenza delle basi, esattamente più alta di 170mila unità la popolazione base di Eurostat proiettata al 1° gennaio 2017, che sottovaluta sia il picco di mortalità del 2015 sia la riduzione delle nascite nel 2015-2016. Negli anni che vanno fino al 2035 tale divario è riassorbito è la differenza tra le popolazioni dei due modelli è pressoché nulla. Nei successivi anni, sotto la spinta delle diverse ipotesi evolutive, particolarmente quelle riguardo ai flussi migratori, il divario cresce fino a 600 mila unità entro il 2045 per arrivare quindi a sfiorare gli 1,8 milioni entro il 2065. Tenuto conto delle differenze metodologiche e della parziale diversità tra le ipotesi, la traiettoria evolutiva della popolazione risulta coerente tra i due scenari.

FIGURA A2. CONFRONTO TRA LA POPOLAZIONE TOTALE PREVISTA NELLO SCENARIO MEDIANO IN BASE 2017 E NELLO SCENARIO EUROSTAT IN BASE 2015. Italia, anni 2015- 2065, milioni.

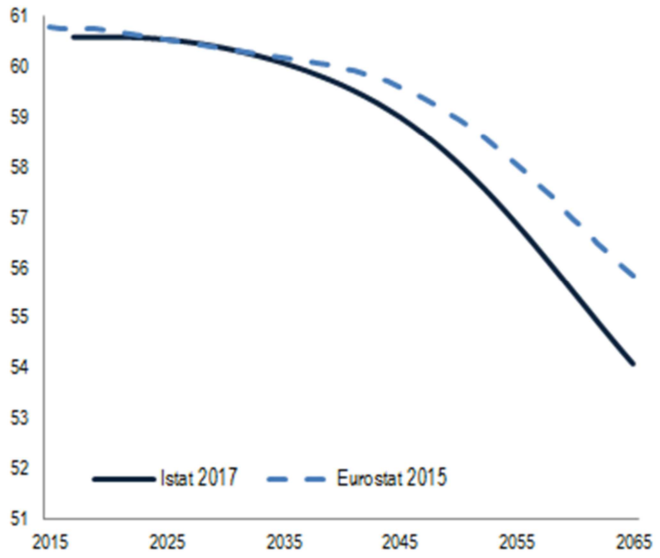

Diffusione dati e termini di utilizzo

Il quadro dettagliato delle ipotesi sottostanti le previsioni e dei principali risultati è consultabile sia sul sito generalista di Istituto dati.istat.it (tema: *Popolazione e famiglie* > *Previsioni demografiche*) sia sul sito tematico demo.istat.it.

La diffusione dei dati è articolata in tre sezioni comprendenti tabelle elaborabili sulla struttura per sesso e singola classe d'età della popolazione, sulle componenti del bilancio demografico e sui principali indicatori demografici. Ciascuna tabella riporta i valori dello scenario mediano e i limiti, inferiore e superiore, degli intervalli di confidenza al 90%, all'80% e al 50%.

Tra le componenti del bilancio sono inclusi:

- ✓ popolazione a inizio e fine anno, saldo totale
- ✓ nascite e decessi, saldo naturale
- ✓ iscritti e cancellati con l'estero, saldo migratorio estero
- ✓ iscritti e cancellati con l'interno, saldo migratorio interno.

I dati sopra descritti e quelli relativi alla distribuzione per età della popolazione sono arrotondati all'unità.

Per quanto riguarda gli indicatori demografici le tabelle comprendono:

- ✓ tassi di natalità, mortalità e crescita naturale
- ✓ tasso immigratorio dall'estero, emigratorio per l'estero e tasso migratorio netto con l'estero
- ✓ tasso immigratorio dall'interno, emigratorio per l'interno e tasso migratorio netto con l'interno
- ✓ tasso migratorio netto totale e tasso di crescita totale
- ✓ età media della popolazione
- ✓ % di popolazione 0-14 anni, 15-64 anni, 65 anni e più, 85 anni e più
- ✓ indici di dipendenza strutturale, di dipendenza degli anziani e di vecchiaia
- ✓ numero medio di figli per donna
- ✓ speranza di vita alla nascita e a 65 anni di età per sesso.

La riproduzione delle informazioni contenute nella presente nota e nelle banche dati dati.istat.it e demo.istat.it è lasciata libera, a condizione che venga citata la fonte Istat.

Riferimenti bibliografici

1. Alho J.M. e Nikeer T. (2004), *Uncertain population of Europe - summary results from a stochastic forecast*. http://www.stat.fi/tup/euupe/rp_reports_e_pub.html.
2. Alho J.M., Spencer B.D. (2005), *Statistical demography and forecasting*. New York: Springer.
3. Bernard A., Bell M. (2012), *A Comparison of Internal Migration Age Profile Smoothing Methods*. Working Paper 2012/01. Queensle Centre for Population Research, The University of Queensle.
4. Billari, F.C., Corsetti G., Graziani R., Marsili M. e Melilli E. (2013), *Towards stochastic forecasts of the Italian population: an experiment with conditional expert elicitations*. Eurostat-Unece Work session on demographic projections, Roma, 29-31 Ottobre 2013.
5. Billari, F.C., Corsetti G., Graziani R., Marsili M. e Melilli E. (2014), *A stochastic multi-regional model for Italian population projections*. Budapest, 25-28 Giugno 2014, European Population Conference. http://epc2014.princeton.edu/papers/140361.
6. Billari, F.C., Graziani R. e Melilli E. (2012), *Stochastic population forecasts based on conditional expert opinions*. Journal of the Royal Statistical Society. Series A. 175(2): 491-511.
7. Booth H. (2006), *Demographic forecasting: 1980 to 2005 in review*, International Journal of Forecasting, 22: 547–581.
8. CBS (2011), *Key figures of the population forecasts 2010-2060*. Statline, Centraal Bureau voor der statistiek, http://statline.cbs.nl/statweb/.
9. Corsetti G., Marsili M. (2012), *A stochastic population projection from the perspective of a national statistical office*. European Population Conference 2012. Stoccolma, 13-16 Giugno 2012, EAPS. http://epc2012.princeton.edu/papers/120635.
10. Corsetti G., Marsili M. (2013), *Previsioni stocastiche della popolazione nell'ottica di un Istituto nazionale di statistica*. Rivista di stastistica ufficiale, n. 2-3, p. 5-29, Istat.
11. Eurostat (2015), *People in the EU: who are we and how do we live? - 2015 edition*, Luxembourg: Publications Office of the European Union.
12. Eurostat (2017), *Population projections at national level*, http://ec.europa.eu/eurostat/.
13. Graziani R., Keilman N. (2011), *The sensitivity of the Scaled Model of Error with respect to the choice of the correlation parameters: A simulation study*. Working Paper 37. Carlo F. Dondena Centre for Research on Social Dynamics, Università Bocconi, Milano.
14. Istat (1989), *Previsioni della popolazione residente per sesso, età e regione – Base 1.1.1988*, Note e Relazioni, n.4.
15. Istat (1997), *Previsioni della popolazione residente per sesso, età e regione – Base 1.1.1996*, Informazioni, n. 34.
16. Istat (2001), *Previsioni della popolazione residente base 1° gennaio 2000*, Statistiche in breve, www.istat.it
17. Istat (2003), *Previsioni della popolazione residente per sesso, età e regione, base 1.1.2001*, Istat, Informazioni n.13.
18. Istat (2006), *Previsioni demografiche nazionali 1° gennaio 2005-1° gennaio 2050*, www.istat.it, Nota informativa, 22 marzo 2006.
19. Istat (2008), *Previsioni demografiche 1° gennaio 2007-1° gennaio 2051*, www.istat.it, Nota Informativa, 19 giugno 2008.
20. Istat (2011), *Il futuro demografico del paese - Previsioni regionali della popolazione residente al 2065*, Statistiche Report, www.istat.it, 28 dicembre 2011.
21. Istat (2017), *Il futuro demografico del paese - Previsioni regionali della popolazione residente al 2065*, Statistiche Report, www.istat.it, 26 aprile 2017.

22. Istat (2018), *Indicatori demografici – stime per l'anno 2017*, Statistiche Report, www.istat.it, 8 febbraio.
23. Keilman N., Pham D.Q. e Hetle A. (2002), *Why population forecasts should be probabilistic - illustrated by the case of Norway*, Demographic Research, 6(15): 409-454.
24. Lee R.D. (1998), *Probabilistic Approaches to Population Forecasting*, Population e Development Review 24, Issue Supplement: Frontiers of Population Forecasting: 156-190.
25. Lee R.D., Carter L.R. (1992), *Modeling and forecasting U.S. Mortality*, Journal of the American Statistical Association, Settembre, vol. 87, n.419.
26. Lee R.D., Miller T. (2001), *Evaluating the performance of the Lee-Carter method for forecasting mortality*, Demography, November, vol. 39, p. 537-549.
27. Lutz, W., Sanderson W.C. e Scherbov S. (1998) *Expert-Based Probabilistic Population Projections*, Population e Development Review, 24: 139-155.
28. Marsili M. (2007), *Demographic projections: the impact of net international migration on population ageing in Italy*, Atti del Convegno Intermedio della SIS 2007 "Rischio e Previsione", Università Ca' Foscari, Venezia, 6-8 giugno.
29. Rogers A. (1985), *Regional Population Projection Models*. Beverly Hills. CA: Sage.
30. Rogers A., Castro L. (1981) *Model migration schedules*, International Institute for Applied System Analysis, Laxenberg, Austria, RR-8 1-30, Novembre 1981.
31. Rowan S., Wright E. (2010), *Developing stochastic population forecasts for the United Kingdom: Progress report e plans for future work*. Eurostat-UNECE Work session on demographic projections, Lisbon, 28-30 Aprile 2010, Methodologies e Working papers, Commissione Europea.
32. Schmertmann C.P. (2003), *A system of model fertility schedules with graphically intuitive parameters*, Demographic Research, 9(5): 81-110.
33. Shaw C. (2008), *The National Population Projections Expert Advisory Group: results from a questionnaire about future trends in fertility, mortality e migration*. Population trends n.134, Winter 2008, Office for national statistics.
34. Stoto, M. A. (1983), *The accuracy of population projections*. Journal of the American Statistical Association. 78: 13–20.
35. Tuljapurkar S., Lee R.D. e Li Q. (2004), *Random scenario forecast versus stochastic forecasts*. International Statistical Review. 72: 185–199.
36. Terra Abrami V. (1998), *Le previsioni demografiche*, Il Mulino, Bologna.
37. United Nations (2015), *World Population Prospects: The 2015 Revision, Methodology of the United Nations Population Estimates and Projections*, Department of Economic and Social Affairs, Population Division, Working Paper No. ESA/P/WP.242.

Glossario

Anagrafe della popolazione: il sistema continuo di registrazione della popolazione residente. Viene continuamente aggiornata tramite iscrizioni per nascita da genitori residenti nel Comune, cancellazioni per morte di residenti e iscrizioni/cancellazioni per trasferimento di residenza da/per altro Comune o da/per l'Estero**.**

Campo di variazione (*range*): misura della variabilità di un fenomeno quantitativo definita dalla differenza tra il valore massimo e il valore minimo osservato.

Crescita naturale (tasso di): la differenza tra il tasso di natalità e il tasso di mortalità.

Crescita totale (tasso di): la somma del tasso migratorio netto totale e il tasso di crescita naturale.

Coorti componenti (modello): l'algoritmo di calcolo continuo che in modalità iterativa simula l'evoluzione dell'equazione fondamentale della popolazione per classi di età, consentendo di determinare le poste demografiche oggetto di previsione e di ottenere la popolazione superstite alla fine di ogni anno.

Decesso: la cessazione di ogni segno di vita in un qualsiasi momento successivo alla nascita vitale.

Dipendenza anziani (indice di): rapporto tra la popolazione di 65 anni e più e la popolazione in età attiva (15-64 anni), moltiplicato per 100.

Dipendenza strutturale (indice di): rapporto tra popolazione in età non attiva (0-14 anni e 65 anni e più) e popolazione in età attiva (15-64 anni), moltiplicato per 100.

Emigratorio interno (tasso): il rapporto tra il numero di cancellati per l'interno e l'ammontare medio della popolazione residente, moltiplicato per 1.000.

Emigratorio per l'estero (tasso): il rapporto tra il numero di cancellati per l'estero e l'ammontare medio della popolazione residente, moltiplicato per 1.000.

Età media: età media della popolazione detenuta a una certa data espressa in anni e decimi di anno.

Età media al parto: l'età media al parto delle madri espressa in anni e decimi di anno, calcolata considerando i soli nati vivi.

Fecondità per età (tasso specifico di): il rapporto tra il numero di nati vivi da donne di età compresa tra x e x+1 e il numero medio di donne residenti di tali età in un dato anno.

Immigratorio dall'estero (tasso): il rapporto tra il numero di iscritti dall'estero e l'ammontare medio della popolazione residente, moltiplicato per 1.000.

Immigratorio interno (tasso): il rapporto tra il numero di iscritti dall'interno e l'ammontare medio della popolazione residente, moltiplicato per 1.000.

Intervallo predittivo (o di confidenza): un intervallo associato a una variabile casuale ancora da osservare, con una specifica probabilità che la variabile casuale vi ricada all'interno.

Iscrizione e cancellazione anagrafica per trasferimento di residenza: l'iscrizione riguarda le persone trasferitesi in un Comune da altri Comuni o dall'estero; la cancellazione riguarda le persone trasferitesi in altro Comune o all'estero.

Migratorio netto con l'estero (tasso): la differenza tra il tasso immigratorio dall'estero e il tasso emigratorio con l'estero.

Migratorio netto con l'interno (tasso): la differenza tra il tasso immigratorio dall'interno e il tasso emigratorio per l'interno.

Migratorio netto totale (tasso): la somma del tasso migratorio netto con l'interno e il tasso migratorio netto con l'estero.

Mortalità (tasso di): rapporto tra il numero dei decessi nell'anno e l'ammontare medio della popolazione residente, moltiplicato per 1.000.

Nato vivo: Il prodotto del concepimento che, una volta espulso o completamente estratto dal corpo materno, indipendentemente dalla durata della gestazione, respiri o manifesti altro segno di vita.

Natalità (tasso di): rapporto tra il numero dei nati vivi dell'anno e l'ammontare medio della popolazione residente, moltiplicato per 1.000.

Numero medio di figli per donna: il numero di figli che una donna metterebbe al mondo nel caso in cui, nel corso nella propria vita riproduttiva, fosse sottoposta al calendario di fecondità (sotto forma di tassi specifici di fecondità per età) dell'anno di osservazione.

Previsione: sviluppo atteso nel futuro.

Previsione demografica: elaborazione che mostra lo sviluppo futuro di una popolazione quando vengono assunte determinate ipotesi riguardo al futuro corso della mortalità, della fecondità e della migratorietà.

Previsione demografica deterministica: elaborazione sul futuro sviluppo di una popolazione, riassumibile in una singola serie di valori ottenuti da un singolo set di ipotesi demografiche, che non riporta alcuna misura riguardo all'incertezza usualmente associabile ai risultati.

Previsione demografica probabilistica: elaborazione sul futuro sviluppo di una popolazione, riassumibile in un set di valori o in una distribuzione di probabilità, in cui le variabili utilizzate sono di natura casuale che non possono essere previste con certezza e in cui non tutte le ipotesi sono egualmente probabili.

Popolazione residente: costituita in ciascun Comune (e analogamente per altre ripartizioni territoriali) delle persone aventi dimora abituale nel Comune stesso. Non cessano di appartenere alla popolazione residente le persone temporaneamente dimoranti, in altro Comune o all'estero, per l'esercizio di occupazioni stagionali o per causa di durata limitata.

Probabilità (prospettiva) di migrazione interregionale: la probabilità che un individuo di età x (in anni compiuti al 1° gennaio) sposti residenza tra due regioni prima che si concluda l'anno.

Probabilità (classica) di morte: la probabilità che un individuo di età precisa x muoia prima del compimento del compleanno x+1.

Probabilità (prospettiva) di morte: la probabilità che un individuo di età x (in anni compiuti al 1° gennaio) non sopravviva entro l'anno.

Saldo migratorio con l'estero: differenza tra il numero degli iscritti per trasferimento di residenza dall'estero e il numero dei cancellati per trasferimento di residenza all'estero.

Saldo migratorio interno: differenza tra il numero degli iscritti per trasferimento di residenza da altro Comune e il numero dei cancellati per trasferimento di residenza in altro Comune.

Saldo migratorio totale: la somma del saldo migratorio con l'estero e del saldo migratorio interno.

Saldo naturale (o dinamica naturale): differenza tra il numero di nascite e il numero di decessi.

Saldo totale: somma del saldo naturale e del saldo migratorio totale.

Scenario previsivo: la descrizione del contesto, anche concettuale, nel quale la popolazione viene proiettata. In un approccio deterministico normalmente si riferisce all'ipotesi definita principale o centrale. In uno stocastico può riferirsi all'ipotesi identificata come media o mediana.

Simulazione: l'implementazione quantitativa di un singolo set di ipotesi demografiche da lanciare nel modello coorti-componenti al fine di ottenere un singolo set di previsioni demografiche.

Speranza di vita alla nascita (o vita media): il numero medio di anni che una persona può contare di vivere dalla nascita nell'ipotesi in cui, nel corso della propria esistenza, fosse sottoposta ai rischi di mortalità per età dell'anno di osservazione.

Speranza di vita all'età "x": il numero medio di anni che una persona di età compiuta "x" può contare di sopravvivere nell'ipotesi in cui, nel corso della successiva esistenza, fosse sottoposta ai rischi di mortalità per età (dall'età "x" in su) dell'anno di osservazione.

Vecchiaia (indice di): rapporto tra la popolazione di 65 anni e più e la popolazione di età 0-14 anni, moltiplicato per 100.
